# Analysing research trends: A descriptive study of abstracts submitted to the African Conference on Emergency Medicine 2024

**DOI:** 10.1016/j.afjem.2025.100934

**Published:** 2025-12-24

**Authors:** Robert Holliman, Peter Hodkinson, Megan Cox

**Affiliations:** a Division of Emergency Medicine, University of Cape Town, South Africa; b Faculty of Medicine and Health, University of Sydney, Australia; c Department of Emergency Medicine, University of Botswana, Botswana

**Keywords:** AfCEM, Research capacity, Authorship trends, African research

## Abstract

**Introduction:**

Emergency care (EC) is rapidly expanding across Africa, yet research output remains limited despite high regional need. The African Conference on Emergency Medicine (AfCEM) is the continent’s only dedicated EC scientific meeting, offering a unique platform to showcase and strengthen regional research. This analysis of abstracts from AfCEM 2024 aims to explore authorship patterns, research themes, and partnerships, highlighting progress and opportunities in African EC research.

**Method:**

Three reviewers analysed the 217 accepted AfCEM 2024 abstracts by authorship, country involvement, partnerships, topic, and methodology. Findings were summarised descriptively and compared with data from previous AfCEM conferences to explore trends in emergency care research across the region.

**Results:**

Of 217 abstracts submitted to AfCEM 2024, East Africa contributed the largest share (43 %), with marked underrepresentation from Central and North Africa. Authorship spanned 34 countries, with 70.5 % of authors and 74.7 % of first authors affiliated with African institutions. However, 54.3 % of abstracts included at least one HIC author, and these were over four times more likely to have five or more contributors. Supadel-sponsored or resident trainees accounted for 16 % of first authors. Most abstracts presented primary data (69.1 %) and used observational or descriptive methods (50.2 %), with 20.3 % employing qualitative or mixed approaches. Research largely focused on emergency presentations, highlighting a growing but uneven research landscape across the continent

**Conclusion:**

This analysis highlights growing African leadership and thematic diversity in EC research, as showcased at AfCEM 2024. While collaborations with high-income countries remain common, African-led work is increasing. Research largely aligns with global priorities but remains predominantly observational, with limited patient-centred or francophone representation. AfCEM continues to reflect and shape the region’s research landscape. Ongoing efforts should focus on equitable partnerships, broader access, and inclusive, cross-disciplinary engagement.

**African Relevance**

• Emergency care research in Africa is evolving with growing local outputs
• The African Conference on Emergency Medicine (AfCEM) is likewise growing and hosted the biggest conference to date in Gaborone in 2024, showcasing the work going on in Emergency Care across the continent.
• AfCEM24 was a leading platform for African authors to share and disseminate work.
• This article reviews all AfCEM24 abstracts, giving a snapshot of Emergency Care research trends

Alt-text: Unlabelled box

## Introduction

Emergency Care (EC) continues to develop in Africa, with postgraduate medical speciality training now offered in over 10 countries [1]. Advances in emergency nursing are occurring throughout the continent, and many African countries, looking towards the World Health Organization (WHO) universal health goals, are developing prehospital care systems [1,2]. Although the critical role of EC is now clear in most health systems internationally, research in the discipline lags, especially in lower-middle income country (LMIC) settings such as Africa, with arguably the highest burden of acute healthcare needs. Meaningful progress in EC within these contexts depends on robust, context-specific research to better understand local needs, challenges, and effective solutions in order to advance the discipline as well as to achieve broader health targets such as the Sustainable Development Goals, and the commitments set out in World Health Assembly Resolution 76.2, which calls for strengthened emergency, critical, and operative care as part of universal health coverage and health emergency preparedness [3].

The African Conference on Emergency Medicine (AfCEM) occurs biennially as the only scientific conference bringing together EC stakeholders from across Africa and the rest of the world [4]. The progress expansion of AfCEM reflects not only the growing scale of the conference, but also the parallel develop of its scientific contributions (Table 1) [[5], [6], [7]].

**Table 1:** Prior AfCEM conferences and participation [[6], [7], [8]].

Conference No.	Year	Location	No. of Attendees	No. of Accepted Research presentations^1^	Number of Supadel sponsored delegates^2^
1	2012	Ghana	439	67 (57 poster, 10 oral)	Data unavailable
2	2014	Ethiopia	498	62 speakers (41 posters 21 oral)	19
3	2016	Egypt	500+	87 speakers (29 posters 58 oral)	36
4	2018	Rwanda	475	105 (58 posters, 47 oral)	23
5	2020	Kenya	Online only	67 abstracts	N/A
6	2022	Ghana	410 hybrid	212 (168 posters, 44 oral)	42
7	2024	Botswana	660+	236 (154 posters, 82 oral)	95

1 Although it would be ideal to make more comparisons on the research presentations at prior AfCEM conferences, this information was not readily available and beyond the scope of this analysis.

2 Supadel is the “Support a delegate” program that the African Federation of Emergency Medicine facilitates, providing financial support to LMIC delegates to attend the conferences.

The 7th AfCEM was held in Gaborone, Botswana, from 6 to 8 November 2024, hosted by the Botswana Society of Emergency Care. The conference theme, *"Working Together for Emergency Care,"* underscored the importance of cross-disciplinary collaboration among clinicians, nurses, and prehospital providers, particularly in the post-pandemic context.

This article presents a secondary analysis of the 217 published abstracts from AfCEM 2024, evaluating authorship patterns, country of origin, research topics, and common methodologies [8]. In addition, we explored the nature of collaborations with high-income countries (HICs) and alternative authorship models to identify emerging trends in EC research. By mapping current outputs and partnerships, this analysis aims to inform future research priorities, promote more equitable collaboration, and support the continued growth of the EC research ecosystem in Africa.

## Methods

In July 2023, the AfCEM2024 scientific committee was established with a collaborative team of researchers in emergency nursing, emergency medicine (EM) and pre-hospital care. This committee utilised the open-access website Oxford abstracts for its scientific platform (Oxford Abstracts Ltd, Oxford, United Kingdom) and placed a first call for abstracts in April 2024. Acceptance of abstracts closed in September 2024, with >270 submissions received. Over 20 EC experts participated in a peer review process, grading abstracts with a minimum of two reviewers per submission. As this was the first onsite AfCEM post-pandemic, the scientific committee aimed to showcase as much African EC research as possible, and were liberal in their acceptance strategies. An initial 81 abstracts were awarded oral presentations and 176 poster presentations, with 40 abstracts withdrawn before the conference (largely due to confirmed non-attendance by the presenting authors). There were thus 217 abstracts presented at AfCEM2024, all of which were published in a special publication in the African Journal of Emergency Medicine (AfJEM) [8]. All abstracts were submitted in English; therefore, it was not necessary to obtain reviews in other languages.

Post AfCEM2024, three independent reviewers analysed the 217 published abstracts under sub-headings related to authorship, country involvement (where identifiable, or noted as not applicable), partnerships, ​​research typology and topic. Abstracts were randomly allocated to one of the three reviewers for data extraction, and reviewed by another reviewer if there was any conflicting information or flagged as unclear. Themes for the research topic as well as the research methodology were developed by consensus, leaning on other publications and general use classifications. Abstracts were also examined for resident (training EM specialist) and Supadel authorship (Support-a-delegate programme), to identify the number of potential first time EC presenters involved at AfCEM2024. The analysed data was compared with published information available on previous conferences, abstract submissions, research topics, authors and priorities for the African region. Data were analysed using Microsoft Excel and presented using standard measures of central tendency.

As this analysis is of already published abstracts, a waiver of ethical approval was attained from the University of Cape Town HREC (HREC 126/2025). All AfCEM2024 abstract submitting authors gave permission for their work to be published as part of the application process.

## Results

**Fig. 1:**
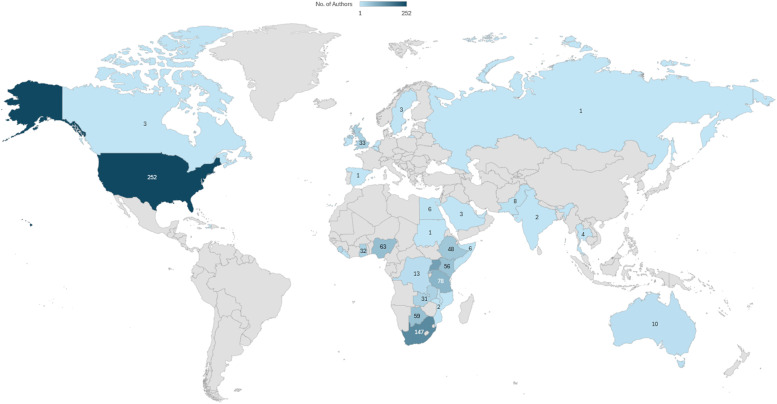
Authorship countries of AfCEM 2024 published abstracts.

A total of 1076 authors were listed across the 217 abstracts submitted to AfCEM2024, representing 34 countries globally (Fig. 1). Of these, 18 countries (52.9 %) were from the African continent. Overall, 747 authors (70.5 %) were affiliated with African institutions. The countries with the highest representation included South Africa (147 authors, 19.7 %), Uganda (111, 14.9 %), Tanzania (78, 10.5 %), Nigeria (63, 8.5 %), Botswana (59, 7.9 %), Kenya (56, 7.5 %), Rwanda (54, 7.2 %), and Ethiopia (48, 6.4 %) (Supplementary Table 1).

Regional representation within Africa was led by East Africa, contributing the highest number of abstracts (94; 43 %), followed by Southern Africa (53; 24 %) and West Africa (33; 15 %). Central and North Africa were markedly underrepresented, with only 3 (1 %) and 2 (1 %) abstracts respectively.

Although difficult to establish with certainty, the cadre of staff described by each abstract referred to EC doctors in 86 (39.6 %) of abstracts, mixed cadres (52; 24 %), prehospital providers (27; 12.4 %), nurses (15; 6.9 %) with 37 (17 %) undetermined. Analysis of the 217 published abstracts showed that 99 (45.6 %) had authorship entirely from LMICs, and therefore 118 (54.3 %) included at least one author affiliated with a HIC (Table 2).

**Table 2:** Mix of authorship countries.

**Single Country Authorship**	**105**	**48%**
Low-and Middle-income Countries (LMIC)*	94	43.3
High-income Countries (HIC)	11	5.1
**Multiple Country Authorship**	**112**	**52%**
Single LMIC + single HIC**	82	37.8
Mix multiple LMIC + HIC	23	10.6
Multiple LMICs	5	2.3
Multiple HICs	2	0.9
**Grand Total**	**217**	

*Including 3 abstracts from LMICs not in Africa (India, Pakistan).

**including 1 abstract from a LMIC not in Africa (Haiti).

The mean number of authors per abstract was 4.9 (range 1–29). Abstracts involving at least one HIC author had both a higher mean (6.3) and median (6) number of contributors compared to those with only LMIC authors (mean 3.4; median 3). This disparity extended to team size also, with only 15 (16 %) of abstracts with exclusively LMIC authors listing five or more contributors, compared to 74 (63.2 %) among those involving at least one HIC author (Table 3).

**Table 3:** Abstracts with High-income Countries authors involved vs Low-and Middle-income Countries authors only.

	HIC involved	LMIC authors only
Number of Abstracts	118 (54.4 %)	99 (45.6 %)
Number of Authors		
Total Mean Median 5 or more Range	741 6.3 6 75 (64 %) 1–19	335 3.4 3 15 (15 %) 1–29

HIC high-income-country; LMIC low-middle-income-country.

**Fig. 2:**
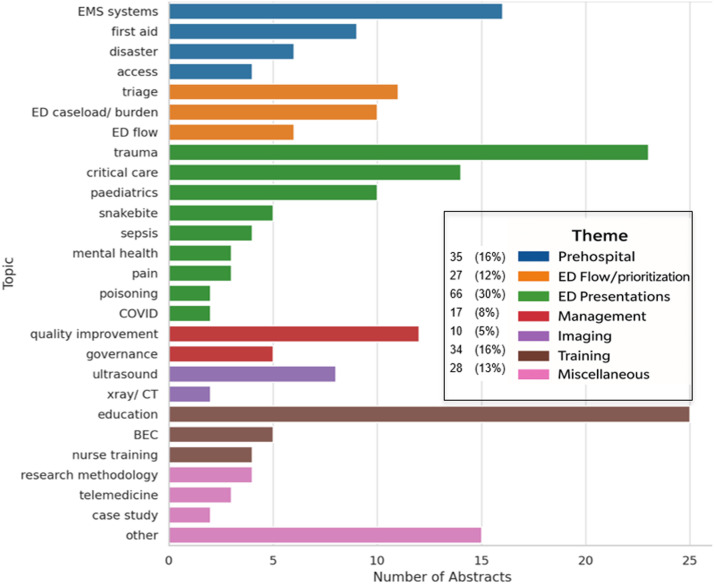
Distribution of AfCEM 2024 Abstracts by Themes and Individual Topics.

Of the 217 first authors, 162 (74.7 %) were affiliated with institutions in Africa. The highest country-level representation among African first authors was from South Africa (33; 20.4 %), Uganda (23; 14.2 %), Tanzania (19; 11.7 %), and Botswana (16; 9.9 %) (Supplementary Table 2). Authors sponsored through the Supadel programme or currently enrolled in emergency medicine residency training accounted for 35 (16 %) of all first authors across the 217 abstracts. A wide range of topics were represented in the abstracts, with the most common topic being related to emergency department presentations (Fig. 2). Primary data was presented in 150 (69.1 %) of all abstracts, with the remainder representing either preliminary data 14; (6.5 %), opinion pieces 36; (16.6 %) or other 16; (7.4 %) with no significant difference in the data type between abstracts with HIC and LMIC authorship.

*BEC = Basic Emergency Care, ED = Emergency Department, EMS = Emergency Medical Services*

**Fig. 3:**
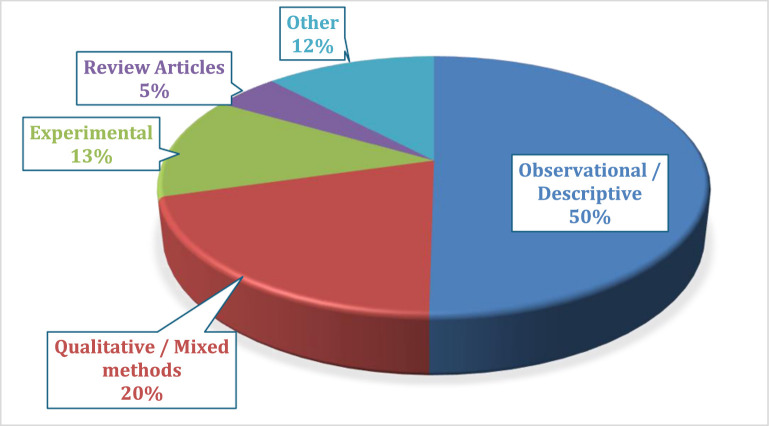
Methodology used in the research.

The most common research methodology was observational or descriptive, featuring in 109 (50.2 %) of the abstracts, and qualitative or mixed methods methodology in 44 (20.3 %) abstracts (Fig. 3).

## Discussion

This review of abstracts submitted to AfCEM2024 highlights the continued expansion of EC research across the African continent, reflecting the broader growth of clinical training programmes, research capacity, and systems development in emergency medicine, nursing, and prehospital care with 217 abstracts presented, represents the highest number in the conference’s history. We believe this upward trajectory suggests a sustained and potentially accelerating growth in African-led or Africa-focused EC research.

Submissions to AfCEM2024 were led by East Africa, followed by Southern Africa and West Africa. This represents a shift from a prior 15 year review of African EC research performed in 2019, which found that EC research output was previously dominated by West Africa (33.5 %) and Southern Africa (32.5 %), with East Africa contributing only 16.6 % [9]. These earlier patterns likely reflected the initial development of EC as a formal specialty in countries such as Ghana and South Africa, where progress was supported by international partnerships at the time [10,11]. The rise in EC research activity from East Africa may be partially influenced by the relative geographic proximity of AfCEM2024, facilitating higher delegate attendance from the region, but likely also reflects the continued maturation of EC systems, training programmes, and academic infrastructure across countries in the region. This progress has been supported by strong regional collaboration; for example, the 2012 East Africa Regional Meeting on Acute and Emergency Care which brought together key stakeholders and helped catalyse joint academic initiatives [12]. Central and North Africa contributed <3 % of abstracts to AfCEM2024, reflecting ongoing under-representation that closely mirrors earlier findings, where the two regions accounted for just 1.7 % and 7.1 % of EC publications, respectively [9].

In Central Africa, limited research activity is likely linked to the absence of established EC systems and nationally recognised training programmes, even in countries like the Democratic Republic of the Congo where emergency units do exist [13]. In North Africa, although health systems are generally better resourced, EC is often delivered through fragmented, specialty-based models again limiting opportunities for EC-specific research outputs [14]. Linguistic barriers may also contribute to regional disparities. Despite efforts to encourage participation, all AfCEM2024 abstracts were submitted in English, potentially disadvantaging francophone countries. While initiatives such as AfJEM’s support for French submissions [15] and the introduction of a francophone stream at AfCEM2024 are welcome, more work is needed to promote inclusive academic engagement across the continent, perhaps with real-time interpretation services at future conferences, as well as dedicated involvement of francophone reviewers and EC collaborators in these areas.

Authorship patterns reflect both progress and ongoing challenges in African EC research, with more than half of presentations including at least one HIC collaborator. Abstracts involving HIC authors had a higher number of contributors than those with only LMIC authors, suggesting a tendency toward larger, more networked teams. This is consistent with a previous analysis from Bruijns et al. [16] who examined African EC publications indexed in Scopus between 2011 and 2015, showing 40 % of publications included authors from outside the continent, while only 12 % involved collaboration between African countries [16]. While HIC partnerships offer important benefits, such as access to research infrastructure, mentorship, and authorship support, they may also reflect a continued dependence on external actors to initiate or sustain research activity. This concern was underscored during the COVID-19 pandemic, when one in five Africa-focused COVID-19 papers had no African authors, and two-thirds of authors on such publications were based outside the continent [17]. In a dynamically shifting global landscape, where established funding channels and geopolitical priorities are increasingly uncertain, such reliance may leave African research efforts vulnerable to external policy changes beyond their control. Strengthening African-led research capacity and regional collaboration is essential to balancing these dynamics and ensuring long-term sustainability [18,19].

A key opportunity to advance this goal lies in supporting early-career researchers and trainees within African EC training programmes. Initiatives such as AFEM’s Supadel programme, designed to facilitate conference participation for those who would otherwise be unable to attend, play a vital role in this regard [20]. At AfCEM2024, 35 of the 217 first-author abstracts (16 %) were submitted by Supadel-sponsored delegates or EC residents in training. Many of these individuals were first-time presenters, and their inclusion reflects the importance of targeted support in expanding access to academic platforms, fostering research confidence, and developing future leaders in EC across the continent.

Topics presented at AfCEM2024 fell predominantly within two of the three overarching domains defined by a recent priority setting review [21]: EC workforce and processes (e.g. nursing and prehospital systems) and EC clinical areas (e.g. trauma). Education, both as a standalone focus and as an integral element of broader systems development, featured prominently across all three of the review’s overarching domains suggesting that researchers presenting at AfCEM2024 are producing work broadly aligned with global EC research priorities. The convergence between AfCEM2024 abstract topics and global priorities could signal either a constructive alignment, or, conversely, a risk of over-reliance on externally driven research priorities at the expense of locally grounded inquiry.

The issue of limited patient focus in EC research has been raised across multiple studies, highlighting a growing concern that research agendas are often shaped solely by expert clinical perspectives [[21], [22], [23]]. More inclusive approaches to priority-setting, incorporating patient and public input, are increasingly recognised as essential to improving the relevance, impact, and efficiency of health research [24]. We found a similar gap in the topics presented at AfCEM2024, where few abstracts addressed patient-centred questions or community-driven priorities. While the emphasis on systems, training, and clinical care remains crucial, greater attention to the patient perspective is needed to ensure that African EC research remains both responsive and accountable to the populations it serves. We found a predominance of exploratory and context-driven studies within the submitted abstracts, most of which were observational or descriptive in nature. This finding is consistent with the earlier review which found that such designs accounted for 85.3 % of African EC publications [9], and with a more recent review by Chironda et al. [25], which reported that 51 % of emergency nursing research in the WHO Afro-region used quantitative methods, most commonly descriptive cross-sectional designs [25]. While EC research in Africa has clearly grown, this trend suggests that the field remains in a formative phase, with a critical need to generate baseline data that captures the structure, performance, and clinical demands of EC systems across the continent, which can help foster local expertise and institutional research culture, and open the doors for more complex research methodologies.

This analysis is based solely on abstracts accepted for presentation at AfCEM2024 and does not represent the full scope of EC research currently conducted across Africa. International conferences such as AfCEM are associated with financial and logistical barriers, particularly for researchers from LMICs. These barriers are especially pronounced for those in nursing and prehospital care, potentially biasing the type of research represented and the groups able to participate. Geographic access also favours research closer to the conference host country. As the largest African EC conference, AfCEM nevertheless provides a meaningful snapshot of research activity among more established or institutionally supported investigators. Another limitation relates to language accessibility. Despite efforts to engage francophone participants, no abstracts were submitted in French. Finally, elements of the analysis, particularly categorisation of topics and methodologies, involved a degree of subjectivity based on reviewer interpretation. To mitigate this, all discrepancies and uncertainties were discussed among the research team and resolved through consensus, ensuring consistent application of coding criteria and maintaining inter-reviewer reliability throughout the analysis.

## Conclusion

This analysis demonstrates the continued growth and diversification of EC research across Africa, as reflected in the abstracts presented at AfCEM2024. There is increasing engagement by African EC professionals, with emerging local leadership and a broad range of research topics. While HIC collaborations remain prominent, African-led work is clearly gaining ground. The predominance of observational methodologies and limited use of experimental designs or systematic reviews suggest that the research environment remains in a developmental phase, with further investment needed to expand methodological depth. Abstract themes largely align with global priorities, though the underrepresentation of patient-centred research and absence of francophone submissions point to areas for more inclusive engagement. As the largest EC conference on the continent, AfCEM provides insight to evolving research capacity and collaboration. Future research must prioritise equitable partnerships, broaden access to academic platforms, and foster cross-disciplinary exchange, ensuring that the spirit of "W*orking* T*ogether* for*Emergency Care"* is matched by meaningful progress across all contexts.

## Dissemination of results

Authors who submitted abstracts to AfCEM2024 will be informed of the publication of this article and its findings. Preliminary results were shared at the International Conference on Emergency Medicine in Montreal, June 2025. We will also seek opportunities to present at future relevant conferences to ensure broad engagement with the EC community.

## Declaration of competing interest

The authors declare no conflicts of interest. It should be noted, however, that PH and RH are editors of AfJEM, but were not involved in editorial process or decisions around this submission. The authors alone are responsible for the views expressed in this article.

## Appendix Supplementary materials

application 1 Image, application 2

## Footnotes

Supplementary material associated with this article can be found, in the online version, at doi:10.1016/j.afjem.2025.100934.
