# Supplementary material for: Analysing research trends: A descriptive study of abstracts submitted to the African Conference on Emergency Medicine 2024
Source: Afr J Emerg Med. 2025 Dec 24;16(1):100934. doi: 10.1016/j.afjem.2025.100934 (PMC12946788; doi:10.1016/j.afjem.2025.100934)
Supplement: application 1 [file mmc1.docx]

## Supplementary Material

Suppl Table 1 Countries of all authors (n=1,076)

| African Country | Number Authors | % Total authors | Non-African Country | Number Authors | % Total authors |
| --- | --- | --- | --- | --- | --- |
| Botswana | 59 | 7,89 | Australia | 10 | 3,13 |
| DRC | 13 | 1,61 | Belgium | 3 | 0.94 |
| Egypt | 6 | 0,80 | Canada | 3 | 0.94 |
| Ethiopia | 48 | 6,29 | Ireland | 3 | 0.94 |
| Ghana | 32 | 4,15 | Netherlands | 1 | 0.31 |
| Kenya | 56 | 7,09 | Russia | 1 | 0.31 |
| Liberia | 8 | 1,07 | Saudi Arabia | 3 | 0.94 |
| Malawi | 4 | 0,54 | Spain | 1 | 0.31 |
| Mozambique | 2 | 0,27 | Sweden | 3 | 0.94 |
| Nigeria | 63 | 8,17 | Thailand | 4 | 1.25 |
| Rwanda | 54 | 7,90 | UAE | 2 | 0.63 |
| Sierra Leone | 26 | 3,75 | UK | 33 | 10.34 |
| Somalia | 6 | 0,80 | USA | 252 | 79.00 |
| South Africa | 147 | 20,88 | Total HIC | 319 | 31,61 |
| Sudan | 1 | 0,13 | Haiti | 2 |  |
| Tanzania | 78 | 10,58 | India | 2 |  |
| Uganda | 111 | 14,86 | Pakistan | 8 |  |
| Zambia | 31 | 3,21 | TOTAL OTHER | 12 | 1,00 |
| Total AFRICA | 745 | 70.5% of total | TOTAL | 1076 |  |

Suppl Table 2 Country of 1^st^ Author

| African Country | First author affiliation | % | Non-African Country | First author affiliation | % |
| --- | --- | --- | --- | --- | --- |
| Botswana | 16 | 9,9 | Australia | 2 | 0,9 |
| DRC | 4 | 2,5 | Belgium | 1 | 0,5 |
| Egypt | 1 | 0,6 | Ireland | 2 | 0,9 |
| Ethiopia | 13 | 8,0 | Netherlands | 1 | 0,5 |
| Ghana | 6 | 3,7 | Sweden | 1 | 0,5 |
| Kenya | 7 | 4,3 | UAE | 1 | 0,5 |
| Liberia | 1 | 0,6 | UK | 8 | 3,7 |
| Malawi | 2 | 1,2 | USA | 36 | 16,6 |
| Mozambique | 1 | 0,6 | India | 1 | 0,5 |
| Nigeria | 15 | 9,3 | Pakistan | 2 | 0,9 |
| Rwanda | 11 | 6,8 | Non-African 1^st^ Author | 55 | 25,3 |
| Sierra Leone | 3 | 1,9 |  |  |  |
| Somalia | 4 | 2,5 |  |  |  |
| South Africa | 33 | 20,4 |  |  |  |
| Sudan | 1 | 0,6 |  |  |  |
| Tanzania | 19 | 11,7 |  |  |  |
| Uganda | 23 | 14,2 |  |  |  |
| Zambia | 2 | 1,2 |  |  |  |
| African 1^st^ Author | 162 | 74,7% |  |  |  |
|  |  |  | Grand Total | 217 |  |
